# SYSTEMI - systemic organ communication in STEMI: design and rationale of a cohort study of patients with ST-segment elevation myocardial infarction

**DOI:** 10.1186/s12872-023-03210-1

**Published:** 2023-05-03

**Authors:** Florian Bönner, Christian Jung, Amin Polzin, Ralf Erkens, Lisa Dannenberg, Rojda Ipek, Madlen Kaldirim, Mareike Cramer, Patricia Wischmann, Oana-Patricia Zaharia, Christian Meyer, Ulrich Flögel, Bodo Levkau, Axel Gödecke, Jens Fischer, Nicolaj Klöcker, Martina Krüger, Michael Roden, Malte Kelm

**Affiliations:** 1 Department of Cardiology, Pulmonology, and Vascular Medicine, Medical Faculty of Heinrich Heine University, University Hospital Düsseldorf, Moorenstr. 5, 40225 Düsseldorf, Germany; 2 Department of Endocrinology and Diabetology, Medical Faculty of Heinrich Heine University, University Hospital Düsseldorf, Düsseldorf, Germany; 3 Institute for Clinical Diabetology, German Diabetes Center, Leibniz Center for Diabetes Research, Düsseldorf, Germany; 4 German Center for Diabetes Research, Partner Düsseldorf, Germany; 5 Departmentn of Cardiology, Evangelisches Krankenhaus Düsseldorf, Düsseldorf, Germany; 6 Experimental Cardiovascular Imaging, Department of Molecular Cardiology, Heinrich Heine University, Düsseldorf, Germany; 7 Cardiovascular Research Institute Düsseldorf (CARID), Medical Faculty of Heinrich Heine University, University Hospital Düsseldorf, Düsseldorf, Germany; 8 Institute for Molecular Medicine III, Heinrich Heine University, Düsseldorf, Germany; 9 Institute for Cardiovascular Physiology, Heinrich Heine University, Düsseldorf, Germany; 10 Institute for Pharmacology and Clinical Pharmacology, Heinrich Heine University, Düsseldorf, Germany; 11 Institute of Neural and Sensory Physiology, Medical Faculty of Heinrich Heine University, University Hospital Düsseldorf, Düsseldorf, Germany

**Keywords:** Master Switches, Metabolism, Myocardial ischemia, STEMI

## Abstract

**Background:**

ST-segment elevation myocardial infarction (STEMI) still causes significant mortality and morbidity despite best-practice revascularization and adjunct medical strategies. Within the STEMI population, there is a spectrum of higher and lower risk patients with respect to major adverse cardiovascular and cerebral events (MACCE) or re-hospitalization due to heart failure. Myocardial and systemic metabolic disorders modulate patient risk in STEMI. Systematic cardiocirculatory and metabolic phenotyping to assess the bidirectional interaction of cardiac and systemic metabolism in myocardial ischemia is lacking.

**Methods:**

Systemic organ communication in STEMI (SYSTEMI) is an all-comer open-end prospective study in STEMI patients > 18 years of age to assess the interaction of cardiac and systemic metabolism in STEMI by systematically collecting data on a regional and systemic level. Primary endpoint will be myocardial function, left ventricular remodelling, myocardial texture and coronary patency at 6 month after STEMI. Secondary endpoint will be all-cause death, MACCE, and re-hospitalisation due to heart failure or revascularisation assessed 12 month after STEMI. The objective of SYSTEMI is to identify metabolic systemic and myocardial master switches that determine primary and secondary endpoints. In SYSTEMI 150–200 patients are expected to be recruited per year. Patient data will be collected at the index event, within 24 h, 5 days as well as 6 and 12 months after STEMI. Data acquisition will be performed in multilayer approaches. Myocardial function will be assessed by using serial cardiac imaging with cineventriculography, echocardiography and cardiovascular magnetic resonance. Myocardial metabolism will be analysed by multi-nuclei magnetic resonance spectroscopy. Systemic metabolism will be approached by serial liquid biopsies and analysed with respect to glucose and lipid metabolism as well as oxygen transport. In summary, SYSTEMI enables a comprehensive data analysis on the levels of organ structure and function alongside hemodynamic, genomic and transcriptomic information to assess cardiac and systemic metabolism.

**Discussion:**

SYSTEMI aims to identify novel metabolic patterns and master-switches in the interaction of cardiac and systemic metabolism to improve diagnostic and therapeutic algorithms in myocardial ischemia for patient-risk assessment and tailored therapy.

**Trial registration:**

Trial Registration Number: NCT03539133

**Supplementary Information:**

The online version contains supplementary material available at 10.1186/s12872-023-03210-1.

## Background

ST-segment elevation myocardial infarction (STEMI) continues to confer a substantial burden of morbidity and mortality with plateauing numbers since 2008 [1, 2]. Latecomer STEMI patients, increased patient age and the associated multimorbidity, and STEMI complicated by cardiogenic shock (CS) counterbalance the improvements in prehospital care, ambulance logistics, pharmacotherapy and timely primary percutaneous coronary intervention (pPCI) [2, 3]. Coronary vessel occlusion by plaque erosion and microembolization have become increasingly frequent compared to plaque rupture, which in turn leads to specific types of myocardial ischemia and infarction [4, 5]. At present, almost one-half of patients still demonstrate left ventricular (LV) postinfarct remodeling [6]. Although there is no difference in long-term survival between remodelers and non-remodelers, LV remodelers experience a higher rate of hospitalization for heart failure [6]. This formulates the need to intensify stratification and therapeutic strategies starting in the acute phase of myocardial ischemia [7]. In elderly STEMI patients, metabolic comorbidities such as type 2 diabetes mellitus (T2DM), anemia, and chronic kidney disease (CKD) become increasingly prevalent. Persons with T2DM exhibit a higher incidence of STEMI and an increased mortality risk [8, 9]. When anemia is added to CKD and T2DM, the prognosis further deteriorates [10]. Of note, T2DM is associated with impaired mitochondrial efficacy not only in skeletal muscle [11] but also in cardiomyocytes [12]. Recently, endotypes (subtypes, clusters) of T2DM with distinct metabolic and inflammatory features have been shown to have adverse cardiovascular risk profiles, particularly among patients with a higher degree of insulin resistance [13, 14]. In parallel, the proportion of STEMI patients without standard modifiable cardiovascular risk factors (SMuRFs) has steadily increased from 14 to 27% in the last decade, particularly among younger individuals, with an almost 50% higher 30-day mortality rate than patients with SMuRFs [15]. This highlights the unmet need to identify unknown confounders in the interactions of systemic and cardiac metabolism. In this regard, systematic cardiocirculatory and metabolic phenotyping to assess the bidirectional interaction of cardiac and systemic metabolism in STEMI and during cardiac healing is lacking.

The **objective** of the present cohort study (systemic organ communication in STEMI, SYSTEMI) is to identify metabolic systemic and myocardial master switches that determine outcomes after STEMI. The aims are as follows: **(i)** to characterize myocardial and systemic metabolism in STEMI together with early regional LV function, LV remodeling, myocardial texture and infarct characteristics at index hospitalization **(ii)** to characterize the interaction of systemic and myocardial master switches using unsupervised cluster analysis to identify patients with a specific metabolic risk phenotype at index hospitalization **(iii)** to identify novel therapeutic master switches and networks prone to interventions and modulations.

## Methods/Design

### Design, setting and endpoints of the study

**Fig. 1:**
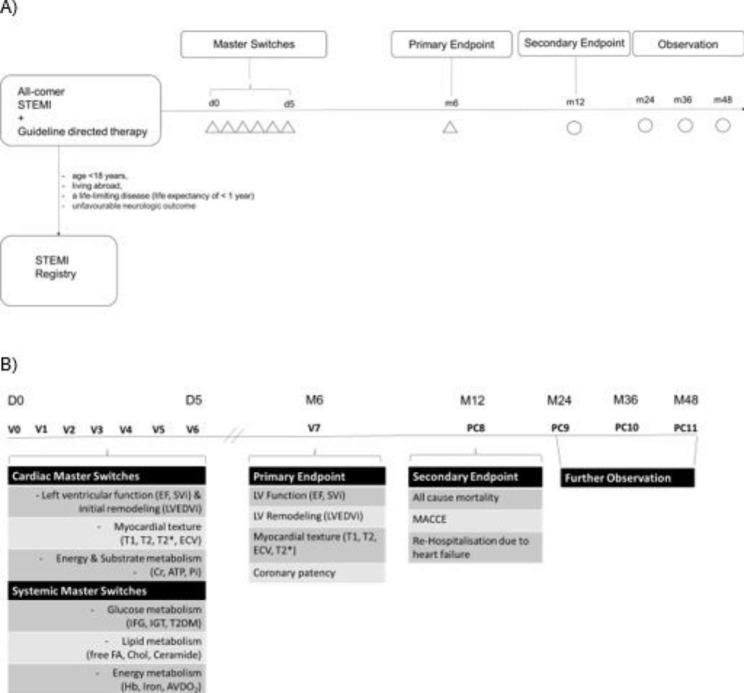
SYSTEMI, systemic organ communication in STEMI, was designed to identify novel metabolic systemic and cardiac masterswitches and networks that determine primary and secondary endpoints, ultimately to improve STEMI care in patient with concomitant metabolic disorders A) Study Flow Chart of SYSTEMI. Patients with STEMI > 18 years of age will be screened and enrolled for study participation if exclusion criteria are not met. Patients fulfilling exclusion criteria are documented within a separate registry. During index hospitalisation 6 Visits (Δ) will be conducted for assessment of myocardial and systemic master switches. At 6 month visit 7 will take place for assessment of the primary endpoints. At 12 month secondary endpoint assessment will be conducted via telephone call while further observation will be conducted yearly. B) Definition of master switches and endpoints. STEMI = ST-segment elevation myocardial infarction, EF = Ejection fraction, SVi = Stroke volume index, WT = Wall thickening, T1 = T1-relaxation time, T2 = T2-relaxation time, ECV = Extracellular volume fraction, Cr = Creatinine, ATP = Adenosine triphosphate, IFG = Impaired Fasting Glucose, IGT = Impaired Glucose tolerance, T2DM = Type 2 Diabetes Mellitus, FA = Fatty acids,

SYSTEMI is an investigator-initiated, open-end prospective, observational, multicentric, cohort study of all-comer patients with STEMI supported by the German Research Council (CRC 1116, Grant No. 236,177,352). Ethics approval was given by the local ethics committees (Heinrich-Heine-University, Düsseldorf, Germany; and Landesärztekammer Nordrhein) complying with the Declaration of Helsinki under reference numbers 5961R and 2,021,055. SYSTEMI is registered at www.clinicaltrials.gov: NCT03539133. SYSTEMI will recruit patients at the academic main-hospital of Heinrich Heine University Düsseldorf, Germany with > 150 STEMI per year, as well as in secondary pPCI -centres within the academic network of Heinrich Heine University Düsseldorf (“Heart-net”), Germany with > 80 STEMI per year. To optimize recruitment efficacy, training sessions have been performed with the interventional staff on call and the study nurses to best inform the patients about the study dependent examinations during their guideline directed diagnostics and therapy at index hospitalisation. As shown in the study flow (Fig. 1A), data will be collected at the index hospitalization (index event within the catheter laboratory + 1–5 days), as well as at follow-up (FU) visits at 6 and 12 months after STEMI at each of the recruiting centers. Follow up phone calls will be performed annually for up to five years. As shown in Fig. 1B, the primary endpoints are imaging-derived LV ejection fraction (LVEF), indexed LV stoke volume (SVi), increase in LV indexed end-diastolic volume (LVEDVi) and LV texture (T1, T2, ECV) using either 3-dimensional echocardiography or CMR 6 months after STEMI as well as angiography derived coronary patency rate at 6 month after STEMI. Secondary endpoint is a composite of all cause mortality, MACCEs (cardiovascular death, nonfatal stroke or myocardial infarction) and rehospitalization due to heart failure within 12 months after STEMI.

### Characteristics of participants

According to our all-comer design, all patients referred to our hospital with a STEMI diagnosis will be asked for informed consent, if no exclusion criterion is present. The main exclusion criteria are age < 18 years, living abroad, and a life-limiting disease with a life expectancy of < 1 year or an unfavourable neurologic outcome. Patients who meet the exclusion criteria will be enrolled in a parallel STEMI register.

Patient selection and short informed consenting will be done by the physician on call at the coronary care unit immediately before the start of pPCI. Before the final consent is given, the investigator or his/her representative will explain verbally the aim, method, source of funding, and the anticipated benefits and potential risks to the participants and answer all questions regarding the study. As part of the general informed consent, all screening subjects are being asked for serial collection of blood samples and its storage in a biobank as well as further genetic analysis. Biosamle processing and storage is done according to existing standard operating procedures (SOPs) at -80 °C. Biosample access is regulated by the SOPs and granted by standardized evaluation upon formal request. Apart from liquid specimen, electronic patient data are collected systematically and online within the Duesseldof Outcome, Safety and Risk Assessment Registry (DOSAR) via health level (HL)-7 interfaces connected to clinical data systems. All requirements of the General Data Protection Regulation are met. A unique participant number will be allocated to each participant (pseudonymization) and assigned chronologically prior to proceeding with study screening. The sequential identification numbers will be used to collect, store, and report participant information.

### Study processes and protocols

The detailed study protocol is given in SPIRIT Table 1. The treatment of STEMI patient will be conducted according to current guidelines [16]. For assessing initial effects of STEMI on the heart and circulation, we will conduct biplane cineventriculography (CVG) as well as right heart catheterisation during pPCI (V0). This will provide information on LV function and circulatory regulation before reperfusion. During right heart catheterization we will measure cardiac output according to the fick`s law, systemic vascular and pulmonary vascular resistance and AVDO_2_ for oxygen consumption. In parallel, the first liquid biopsies will be generated. The liquid biopsies will serve for assessing basic laboratory parameters including lactate to assess cardiogenic shock and biobanking specimen aiming at subsequent cellular, biochemical, immunological and genetic analyses. Guideline directed repetitive ECG recordings and monitoring will be conducted (V1).

**Table 1:** SPIRIT (Standard Protocol Items: Recommendations for Interventional Trials) figure showing important events and their respective time points during the study period in the cohort study

Action	Study Periode											
	Enrolment	Observation										
		Index Hospitalisation						FU Hospitali-sation	FU Call			
Visit (V) / Phone Call (PC)	Screening	V1	V2	V3	V4	V5	V6	V7	PC8	PC9	PC10	P111
Time Point (d)	0	0	1	2	3	4	5	180	365	730	1095	1460
*Enrolment*												
Eligability Screen	X											
Informed Consent	X											
Pseudonymisation	X											
*Assessments*												
Mail-Reporting (internal)		X	X	X	X	X	X	X				
Questionnaires & PE			X	X	X	X	X	X				
CA		X						X				
LHC/RHC		X						X				
CVG		X						X				
TTE			X		X			X				
CMR			X		X			X				
CMR + MRS							X	X				
Tonometry							X	X				
FMD							X	X				
4D Flow CMR							X	X				
12-lead ECG		X	X	X			X	X				
Monitoring		X	X	X								
Blood Sampling & Biobanking		X	X				X	X				
OGTT							X	X				
*Outcome Assessment*												
Endpoints								P	S	O	O	O

PE = Patient Empowerment, CA = Coronary Angiography, LHC = Left Heart Catheterisation, RHC = Right Heart atheterisation, CVG = Cineventriculography, TTE = Transthoracic echocardiography, CMR = Cardiovascular Magnetic Resonance, MRS = Magnetic Resonance spectroscopy, FMD = Flow Mediated Dillatation, ECG = Electrocardiogram, OGTT = oral glucose tolerance test, P = Primary Endpoint, S = Secondary Endpoint, O = Observational

Within the first and third day after reperfusion we will conduct additional cardiac imaging with transthoracic echocardiography and in a subset of patients cardiovascular magnetic resonance (CMR) (1.5 & 3.0T Achieva, PHILIPS Healthcare, Best, Netherlands) including measurements of ventricular mass, volume, function and myocardial relaxometry (V2 + V4). Cine imaging will be used to calculate pressure-volume curves by taking phase-to-phase volumes of short-axis slices, blood pressure and left ventricular end-diastolic pressure measurements from acute catheterization measurements [17]. Additionally, native relaxometric mapping of the myocardium (T1, T2, T2*) [18], gadolinium contrast-enhanced imaging and postcontrast relaxometric mapping (T1, extracellular volume fraction, ECV) will be performed to assess the myocardial inflammation/edema extent, extracellular volume, fibrosis and iron content. For this, validated sequences will be used as outlined in Additional File Table 1. Gadolinium-enhanced images will be used to assess markers of infarcted tissue, such as edema, infarct size (IS), microvascular obstruction (MVO), and intramyocardial hemorrhage (IMH). Certified CMR evaluation software will be used for all analyses (CVI42, Circle Cardiovascular Imaging Inc., Calgary, Alberta, Canada or Sectra Workstation IDS7, Version 19.3.6.3510). This will be accompanied by an additional liquid biopsy. These early measurements after reperfusion will give insight in very early adaptations in ventricular volume and dimensions, which will serve to associate ventricular volume and dimension with systemic blood derived markers. Blood samples will also be collected to assess arterial or venous lactate, hemoglobin A1c (HbA1c), fasting blood glucose and insulin for calculating the Homeostasis Model Assessment indices (HOMA-IR and HOMA-B). Additionally, a detailed assessment of anemia will be performed, including parameters of RBC turnover, formation, (dys)function, and hemolysis. Specifically, we will address absolute and relative iron deficiency (ID), one of the most common reasons of anemia, especially in elderly patients with comorbidities, by measuring free iron, ferritin, transferrin, the soluble transferrin receptor and transferrin saturation in the collected blood samples. This will be done during V2.

During V2-V5 we will additionally empower the patients to stick to the study by providing detailed information about his health status and study specific examination results. Five days after pPCI (V6), we will again apply echocardiography and CMR with the addition of magnetic resonance spectroscopy (MRS). For regional myocardial metabolic profiling, ^1^ H and ^31^P-MRS, which are key techniques for measuring fatty acids (FA) and total creatine as well as ATP, Pi and pH, will be integrated into the clinical CMR protocol 5 days and 6 months after STEMI. For ^1^ H-MRS, navigator-free metabolite-cycled spectroscopy using a voxel size of approximately 4–6 ml (10 × 15 × 35 mm3) and ≈ 144 averages (7:50 min) will be applied [19]. jMRUI/AMARES will also be used to analyze ^31^P spectra. Reconstruction will be performed using a customized reconstruction pipeline in ReconFrame (GyroTools LLC, Zürich, Switzerland). jMRUI/AMARES will also be used to analyze MR spectra. Simultaneously, we will have information through liquid biopsies on the diabetes endotype in patients with overt diabetes and on glucose tolerance in patients without diabetes by applying oral glucose tolerance test (OGTT). To asses circulatory adaptations of large conductive arteries, we will apply pulse wave velocity measurements by 4-dimensional velocity-encoding in CMR and by carotid-femoral applanation tonometry using Sphygmocor® technology (AtCor Medical, West Ryde, Australia). The central aortic blood pressure, pulse pressure (PP) and augmentation index (AIX) will be assessed. To assess the primary endpoints, that is LV function, LV remodeling, LV metabolism, LV texture and coronary patency, after complete myocardial healing at 6 months after STEMI, we scheduled further examinations (V7). These examinations comprise of a coronary angiography, echocardiography, CMR, OGTT, applanation tonometry and additional liquid biopsies. The secondary endpoints will be assessed by a telephone call at 12 months (PC8). Further observational phone calls (PC9-11) are planned on a yearly basis.

### Study organisation and leadership

The SYSTEMI Steering Committee is responsible for the scientific content of the protocol, oversees the study steps, and checks adherence to “Good Clinical Practice” and the study protocol as well as performance. Study conduction will follow the SOP of the Clinical Trial Unit (CTU) of the University Hospital of Düsseldorf. The SYSTEMI Endpoint Adjudication Committee (EAC) will adjudicate clinical endpoints based on data provided by the clinical trial sites. This procedure will be supervised by the trial coordination center (TCC, Koordinierungszentrum für Klinische Studien, KKS) at Heinrich Heine University. The trial management committee (TMC) meets once a week and the steering committee twice a year to review trial conduct. The data monitoring committee (DMC) consist of a data manager and participants of the external advisory board. Patient data are collected systematically and online within the DOSAR Registry via health level (HL)-7 interfaces connected to clinical data systems. All requirements of the General Data Protection Regulation are met. The DMC will report in a weekly manner organizational aspects of data collection and handling as well as quality and completeness reports. Data quality and completeness is of prime importance in SYSTEMI. Regular audits are conducted synchronized to the annual meeting of the Cardiovascular Research Institute Düsseldorf (CARID). Upon formal request according to our internal SOP, access to primary data can be given. This process is guided by the SYSTEMI Steering Committee, which is responsible for the scientific content of the study data.

### Statistical approach

SYSTEMI was designed as open-end prospective cohort study in all comer STEMI patients. No formal sample size calculation has been performed. It was anticipated that more than 160 STEMI patients per year will be a realistic recruitment speed on the basis of expected STEMI patients at host institutions to reach a total sample size of > 1000 STEMI patients.

This cohort study was designed to unravel novel master switches and markers of metabolic networks in STEMI patients to further facilitate risk stratification and identify novel therapeutic targets in STEMI. For primary endpoints, logistic regression analysis will be used to model the predefined outcome data with impact of covariates. The effects of the independent parameters in these regression models will be estimated (odds ratio, hazard ratio) and presented with 95% confidence intervals. Network analysis using machine learning approaches for unsupervised metabolic cluster identification will be conducted. Secondary endpoint analysis will be performed using Kaplan–Meier plots and the log-rank test. Two-tailed p values below 0.05 will be considered indicative of statistically significant differences. Since significant interactions of master switches within the metabolic network are anticipated, interim analysis will be done after 500 and 1000 patients for a robust statistical power calculation and re-adjustment of investigations.

## Discussion

Mortality and morbidity after STEMI remain high and current risk prediction models do not take metabolic risk constellations into account. Therefore, the overall objective of SYSTEMI is to identify systemic and myocardial metabolic master switches that determine outcomes after STEMI. To reach this objective, we have designed and set-up SYSTEMI to comprehensively investigate systemic and myocardial metabolism at index hospitalization and at 6 months in more than 1000 patients. SYSTEMI will enable in-depth analyses of systemic metabolic effectors on local myocardial function and metabolism, as detected by magnetic resonance imaging and spectroscopy, and clinical outcome.

### SYSTEMI metabolic clusters

To our knowledge, the present cohort study is the first study in patients with STEMI to systematically and simultaneously assess organ-function, inter-organ communication as well as myocardial and systemic metabolism. SYSTEMI focuses on 3 metabolic clusters: (i) the glucometabolic spectrum, (ii) the lipid metabolism and (iii) the O_2_ transport capacity of the blood. The first metabolic cluster covers the glucometabolic spectrum of hyperglycemia, hyperinsulinemia, and insulin resistance to T2DM and the role of specific endotypes of T2DM in patients with STEMI. Altered glucose metabolism can be seen in up to 50% of all STEMI patients, but only 25% remain in this state after 3 months [20]. Moreover, the hyperglycemic status of diabetic and nondiabetic patients has been shown to influence IS, MVO and LVEF post-STEMI [20]. Therefore, SYSTEMI will focus on glucometabolic phenotyping using various methods, including OGTT, homeostatic model assessment, or endotyping of T2DM. Endotyping of T2DM has shown different prevalence rates of diabetes-related complications, although their impact on STEMI patients remains unknown [13, 14]. In this context, the severe insulin-resistant diabetes (SIRD) endotype may specifically predispose STEMI patients to metabolic and myocardial alterations. SIRD has been associated with elevated levels of biomarkers of inflammation and a higher risk of diabetic kidney disease [13, 14]. This characterization may help to guide targeted therapeutic regimens for patients with STEMI and T2DM. The recent introduction of sodium-glucose cotransporter-2 inhibitors into guidelines for heart failure in patients with and without T2DM has highlighted the importance of glucometabolic modulation in cardiovascular disease to improve patient outcomes [21]. The second metabolic cluster covers the crosstalk of cardiac ischemia with changes in lipid metabolism in the presence and absence of alterations of the glucometabolic spectrum. The impacts of browning adipose tissue [22], the lipolytic response in skeletal muscle, and circulating lipoproteins, ceramides, and the HDL sphingolipidome [23] on infarct will be investigated. The third metabolic cluster covers function and dysfunction of red blood cells in anemia [24]. Anemia affects systemic energy metabolism through multiple mechanisms, including limited oxygen transport capacity, disturbed iron metabolism, and altered hepatic metabolism, and is associated with profound red blood cell (RBC) dysfunction [24]. This appears to be an important modulator of interorgan crosstalk after STEMI. The prognosis of STEMI is limited by anemia and secondary complications such as bleeding, thrombosis, inflammation, and arrhythmia [25].

### SYSTEMI approaches to characterize myocardial and systemic metabolism

To enable comprehensive investigation of the bidirectional interaction of systemic and cardiac metabolism through the aforementioned pathways in the acute phase of STEMI and its long-term outcome in patients, several layers of analysis have to be accomplished within SYSTEMI: (i) analysis of cardiac and circulatory function prior to and immediately after pPCI, on the 1st, 3rd, and 5th day of the index hospitalization using novel CMR techniques, (ii) assessment of glucose and lipid metabolism, and (iii) readouts of various organ and tissue functions during and after STEMI, including the liver, the pancreas, the spleen, and circulating blood and immune cells arising from bone marrow, the kidney, skeletal muscle, and adipose tissue. This is complemented by targeted multiomics analysis, including proteomics, lipidomics, and long-read sequencing, and novel CMR-based techniques to study myocardial flux rates and energy metabolism in mice and patients. In perspective, SYSTEMI seeks to link regional functional and structural alterations with local myocardial metabolism. Several technical improvements in recent years have facilitated the application of MRS, ^1^ H- and ^31^P-MRS measurements by reducing the scan time or voxel size for examining metabolism in infarcted and remote zones [19]. In experimental models of myocardial ischemia, substrate selection is characterized by a “metabolic switch” in myocardial energy supply from predominantly aerobic FA oxidation to anaerobic glycolysis. ^1^ H-MRS may estimate myocardial triglyceride and total creatine levels, whereas ^31^P-MRS is able to quantify metabolites such as phosphocreatine, ATP, their ratio or creatine kinase fluxes [26]. Currently, data are lacking for CMR/MRS measurements that comprehensively characterize metabolic alterations in ischemic and remote myocardium with respect to coronary artery disease severity using ^1^ H- and ^31^P-MRS. In perspective, myocardial metabolism will be further assessed by use of hyperpolarized ^13^ C-substrates, such as pyruvate, bicarbonate or lactate, combined with advanced deuterium metabolic imaging [27].

## Conclusion

SYSTEMI aims to identify individuals with distinct susceptibility to myocardial ischemia among the different endotypes of T2DM across the glucometabolic spectrum, endotypes of anemia and lipid disorders, paving the way for precision medicine in STEMI and cardiometabolic disorders.

## Electronic supplementary material

Below is the link to the electronic supplementary material.

Additional File Table 1: CMR sequences used and respective references

## Data Availability

The datasets generated during and analyzed during the current study are not publicly available due to the fact that the trial is still recruiting and preliminary results not published in peer reviewed journals but are available from the corresponding author on reasonable request.

## Abbreviations

AI: Artificial intelligence
CEST: Chemical exchange saturation transfer
CS: Cardiogenic shock
CKD: Chronic kidney disease
CMR: Cardiac magnetic resonance
CRC: Collaborative Research Consortium
DMC: Data Monitoring Committee
DOSAR: Düsseldorf outcome and safety registry
EAC: Endpoint Adjunction Committee
EF: Ejection fraction
ECV: Extracellular volume fraction
EDV: End-diastolic volume
ESV: End-systolic volume
FMD: Flow-mediated dilatation
FU: Follow-up
HbA1c: Hemoglobin A1c
HOMA: Homeostasis model assessment
IMH: Intramyocardial hemorrhage
ID: Iron deficiency
IS: Infarct size
LAS: Large artery stiffness
LDL-C: Low-density lipoprotein cholesterol
LV: Left ventricle
MACEs: Major adverse cardiovascular events
MRS: Magnetic resonance spectroscopy
MVO: Microvascular obstruction
6MWT: 6 min walk test
NAFLD: Non-alcoholic fatty liver disease
OGTT: Oral glucose tolerance test
pPCI: Primary percutaneous coronary intervention
PWV: Pulse wave velocity
SIRD: Severe insulin-resistant diabetes
SMuRF: Standard modifiable cardiovascular risk factor
SOP: Standard operating procedure
STEMI: ST-segment elevation myocardial infarction
SV: Stroke volume
SYSTEMI: Systemic organ communication in STEMI
TMC: Trial Management Committee
T2DM: Type 2 diabetes mellitus
