# Supplementary material for: SYSTEMI - systemic organ communication in STEMI: design and rationale of a cohort study of patients with ST-segment elevation myocardial infarction
Source: BMC Cardiovasc Disord. 2023 May 3;23:232. doi: 10.1186/s12872-023-03210-1 (PMC10158247; doi:10.1186/s12872-023-03210-1)
Supplement: Supplementary file 1 — Additional File Table 1: CMR sequences used and respective references [file 12872_2023_3210_MOESM1_ESM.docx]

Supplementary Table S1. CMR sequences used and respective references

| **CMR Sequences** | | |
| --- | --- | --- |
| Item | Technique | Sequence + DOI |
| Volumes, global and local function, myocardial mass | Cine in 4-,3-2ChV, and SA | SSFP  repetition time (TR)/echo time (TE) = shortest, flip angle (FA) = 60°, reconstructed voxel size = 1.46 × 1.45 × 8 mm3, end-expiratory breath-hold  https://doi.org/10.1186/s12968-015-0118-0 |
| Oedema | T2w-imaging  T2-mapping | GRaSE  TR = 1 RR interval, number of echo images = 15, echo spacing 10 ms, leading to an echo train of 150 ms, number of gradient echoes for segmented acquisition = 3 (EPI factor), FA = 90°, spatial resolution: 2 × 2 × 10 mm3, parallel imaging (SENSE) acceleration factor of 2, k-space data acquired with cartesian encoding scheme. Double inversion black-blood pulse  https://doi.org/10.1186/s12968-015-0118-0 |
| Haemorrhage | T2*-mapping | FFE  TR shortest, TE first: short, number of echo images = 6, echo spacing shortest, FA = 20°, spatial resolution 2 × 2 × 8 mm3, parallel imaging (SENSE), fast imaging: TFE, TFE pre-pulse black blood  https://doi.org/11.2234/s32345-232-34-432 |
| Fibrosis | T1-mapping  ECV-mapping | MOLLI  Balanced steady state free precession single breath-hold modified Look-Locker Imaging (MOLLI, (3(3)3(3)5)) (TE/TR/flip-angle: 1.64msec/3.3msec/50°, acquired voxel size 1.8x1.8x8 mm, phase encoding steps n=166, 11 images corresponding to different inversion times (3+3+5 MOLLI scheme), adiabatic prepulse to achieve complete inversion)  https://doi.org/10.1016/j.ejrad.2016.10.031. |
| Cardiac and thoraxic fat volume | mDIXON | FFE  TR shortest, TE first: short, number of echo images = 2, echo spacing shortest, FA = 15°, spatial resolution 1.25 × 1.5 × 1.5 mm3, 100 slices, parallel imaging (SENSE), fast imaging: TFE, TFE pre-pulse no  https://doi.org/10.1007/s00330-020-07517-x |
| Myocardial triglycerides | ^1^H Spectroscopy | PRESS  minimum TR 1 beat, TE shortest, FA = 90°, metabolite cycling by adding an optimized Hwang pulse in front of the sequence, bandwidth 2000 Hz (1024 samples), voxel size from 5 x 10 x 25 mm, pencil beam volume shimming during a single breathold, triggering to maximum systole, 144 averages, free breathing acquisition |
| Vascular Function | 4D Flow | FFE  TR shortest, TE first: short, number of echo images = 1, FA = 5.2°, spatial resolution 2.8 × 2.8 × 2.8 mm3, 40 slices, parallel imaging (SENSE), fast imaging: TFE, TFE pre-pulse no |
| Microvascular patency | Fist Pass Perfusion  (gadoteridol, ProHance®, Bracco Imaging, total dose = 0.2 mmol/kgKG) | FFE  TR shortest, TE shortest, number of echo images = 1, FA = 50°, spatial resolution 3 × 3 × 10 mm3, 3 slices, parallel imaging (SENSE), fast imaging: TFE, TFE pre-pulse saturate  https://doi.org/10.1186/s12968-015-0118-0 |
| Infarct Size | Late Gadolinium enhancement (10 Minutes)  (gadoteridol, ProHance®, Bracco Imaging, total dose = 0.2 mmol/kgKG) | Turbo FFE  TR shortest, TE shortest, 3D gradient spoiled, 180° inversion, FA = 15°, °, spatial resolution 1.52 × 1.71 × 10 mm3, reconstructed voxel size = 1.52 × 1.52 × 5 mm3, end-diastolic, end-expiratory breath-hold  https://doi.org/10.1186/s12968-015-0118-0 |
| Microvascular Obstruction | Late Gadolinium enhancement (5 & 15 Minutes)  (gadoteridol, ProHance®, Bracco Imaging, total dose = 0.2 mmol/kgKG) | Turbo FFE  TR shortest, TE shortest, 3D gradient spoiled, 180° inversion, FA = 15°, °, spatial resolution 1.52 × 1.71 × 10 mm3, reconstructed voxel size = 1.52 × 1.52 × 5 mm3, end-diastolic, end-expiratory breath-hold  https://doi.org/10.1186/s12968-015-0118-0 |
